# The feasibility and clinical effects of dendritic cell-based immunotherapy targeting synthesized peptides for recurrent ovarian cancer

**DOI:** 10.1186/1757-2215-7-48

**Published:** 2014-05-07

**Authors:** Masanori Kobayashi, Asako Chiba, Hiromi Izawa, Eri Yanagida, Masato Okamoto, Shigetaka Shimodaira, Yoshikazu Yonemitsu, Yuta Shibamoto, Noboru Suzuki, Masaki Nagaya

**Affiliations:** 1 Seren Clinic Nagoya, Isokai, 4-14-2 Sakae, Naka-ku, Nagoya 460-0008, Japan; 2 Seren Clinic Tokyo, Isokai, 2-10-2 Shirokanedai, Minato-ku, Tokyo 108-0071, Japan; 3 Institute for Advanced Medical Research, Keio University School of Medicine, 35 Shinanomachi Shinjuku, Tokyo 160-8582, Japan; 4 Cell Processing Center, Shinshu University Hospital, 3-1-1 Asahi, Matsumoto, Nagano 390-8621, Japan; 5 R&D Laboratory for Innovative Biotherapeutics, Graduate School of Pharmaceutical Sciences, Kyushu University, 3-1-1 Maidashi, Higashi-ku, Fukuoka 812-8582, Japan; 6 Department of Radiology, Nagoya City University Graduate School of Medical Sciences, 1 Kawasumi, Mizuho-cho, Mizuho-ku, Nagoya 467-8601, Japan; 7 Department of Immunology, St. Marianna University School of Medicine, 2-16-1 Sugao Miyamae-ku, Kawasaki 261-8511, Japan

**Keywords:** Dendritic cell, WT1, MUC1, CA125, Immunotherapy and recurrent ovarian cancer

## Abstract

**Background:**

Despite the increased rate of complete response to initial chemotherapy, most patients with advanced ovarian cancer relapse and succumb to progressive disease. Dendritic cell (DC)-based immunotherapy has been developed as a novel strategy for generating antitumor immunity as part of cancer treatments. The present study aimed to assess the feasibility and clinical effects of DC therapy for recurrent ovarian cancer (ROC).

**Methods:**

This retrospective study included 56 ROC patients who initially received standard chemotherapy followed by DC-based immunotherapy targeting synthesized peptides at 2 institutions between March 2007 and August 2013. The adverse events (AEs) and clinical responses were examined.

**Results:**

No serious treatment-related AEs were observed. Seventy one percent of the enrolled patients developed an immunologic response. The median survival time (MST) from ROC diagnosis was 30.4 months, and that from the first vaccination was 14.5 months. Albumin levels of ≥4.0 g/dL and lactate dehydrogenase levels of <200 IU/L before vaccination were identified as significant independent factors by multivariate Cox proportional hazard analysis. The MST from the first vaccination in patients with albumin levels of ≥4.0 and <4.0 g/dL were 19.9 and 11.6 months, respectively. The corresponding disease control rates were 36% and 15%, respectively.

**Conclusions:**

Our results demonstrated the feasibility and potential clinical effectiveness of DC-based immunotherapy for ROC patients. Additionally, a good nutritional status might be an important factor for further clinical effects.

## Introduction

According to a 2012 estimate, approximately 22,280 new cases of ovarian cancer (OC) are diagnosed and 15,500 women die of the disease in the United States annually [1]. OC is often detected when the disease is already widespread in the abdomen, with approximately 40–50% of all patients being diagnosed with stage III or IV disease [2]. The standard approach to OC treatment is debulking surgery, followed by combined platinum and taxane chemotherapy [3,4]. Although significant progress in OC treatment has been achieved, approximately 55% of all patients develop recurrence within 2 years, and more than 70% of patients show recurrence within 5 years [2]. Patients with recurrent disease often receive additional second- and third-line chemotherapy regimens. In cases of resistance to platinum-based therapy, second-line single-agent chemotherapy with non-platinum drugs is offered, with a short-lived response rate of approximately 10–25%, regardless of drug types. Combination therapies have been suggested to offer no clinical benefits in these patients [5]. Most patients therefore receive only palliative care, indicating an urgent need for alternative approaches that could improve the survival rates of patients with recurrent ovarian cancer (ROC).

Dendritic cells (DCs) are critical antigen-presenting cells that are characterized by their efficient presentation of internalized antigens with major histocompatibility complexes (MHCs), required to orchestrate T-cell responses [6]. The first DC vaccination study was reported in 1996 [7], and a few clinical trials of DC-based immunotherapy for OC have been conducted [8-10]. However, these trials included patients with all stages of OC and had small sample sizes, thus making it difficult to accurately assess the efficacy of DC immunotherapy in ROC.

In 2009, the cancer antigen prioritization project of the National Cancer Institute ranked Wilms tumor 1 (WT1) as the first antigen, followed by mucin 1, cell-surface associated (MUC1) [11]. The oncogenic WT1 is expressed in various types of hematological and solid malignancies, including OC. The expression frequency of WT1 in OC ranges from 62% to 78% in immunohistochemistry (IHC) studies [12,13].

MUC1 is a heavily glycosylated membrane glycoprotein with 5 potential *O*-glycosylation sites in each of its 20 amino acid-long tandem repeats, which comprise most of the extracellular domain [14]. Numerous studies have shown that MUC1 is widely expressed on carcinomas, including those of the breast, colon, rectum, stomach, and lung, as well as OC [15]. Wang et al. [16] reported that MUC1 expression was detected by IHC in 90% of OC cases. Additionally, cancer antigen (CA)-125 has also been detected in tissue sections of human epithelial OC, but not in normal ovarian tissue. Høgdall et al. [17] confirmed that CA125 expression was observed in 70% of all examined OC tissue samples.

Although WT1, MUC1, and CA125 are considered tumor antigens and potential targets for cancer immunotherapy, no reports are available on these antigens in ROC. Therefore, we retrospectively investigated the safety, immunological responses, and clinical effects of DC vaccines targeting synthesized peptides in ROC patients.

## Materials and methods

### Patient selection

This retrospective study included patients who initially received chemotherapy for ROC followed by DC-based immunotherapy targeting synthesized peptides at the Seren Clinics in Nagoya and Tokyo between March 2007 and August 2013. The inclusion criteria were as follows: (1) a clinical diagnosis of inoperable ROC; (2) an expected prognosis of more than 3 months; (3) white blood cell count of 2,000 cells/μL or higher; (4) hemoglobin level of 7.0 g/dL or higher; (5) platelet count of 70,000 counts/μL or higher, and (6) no serious vital organ dysfunction. All participants provided signed informed consent for use of their data for this study. This study was approved by the institutional review board of Isoukai (approval number 25–2) and was conducted in accordance with the Declaration of Helsinki.

### DC preparation

To determine the type of peptides for administration, we first evaluated each patient for human leukocyte antigen (HLA) expression. In cases of available patient tissue samples, WT1 and/or MUC1 expression was assessed via IHC staining. Serum CA125 levels were also evaluated to determine the peptide to be administered. Based on these results, DCs were prepared and pulsed with 1–3 synthesized peptides as previously described [18,19]. Briefly, peripheral blood mononuclear cells (PBMCs) were prepared from leukapheresis products by Ficoll-Hypaque gradient density centrifugation. PBMCs were then plated on tissue culture vessels and continuously cultured for 5 days in medium containing granulocyte-monocyte colony-stimulating factor (GM-CSF; 50 ng/mL) and interleukin (IL) 4 (25 ng/mL) to generate immature DCs. To induce further differentiation, the immature DCs were stimulated with OK-432 (Chugai Pharmaceutical Co, Ltd, Tokyo, Japan) and prostaglandin-E2 (50 ng/mL; Daiichi Fine Chemical Co, Ltd, Toyama, Japan) for 24 hours. On day 7 in culture, the DCs were pulsed with MHC class I-restricted WT1 peptide antigens according to the HLA-A pattern (CYTWNQMNL [mutant WT1 peptide; Neo-MPS, San Diego, CA] for HLA-A*2402 or RMFPNAPYL [WT1 peptide; Neo-MPS] for HLA-A*0201/0206), MUC1 long peptide (30-mer [TRPAPGSTAPPAHGVTSAPDTRPAP-GSTAP] at 20 mg/mL; Greiner Japan, Tokyo, Japan) for any HLA-A type, and/or CA125 protein (500 U/mL) for any HLA-A type. Subsequently, the DCs were characterized by flow cytometry to ensure that they achieved the typical phenotype of mature DCs (CD14^-/low^/HLA-DR^+^/HLA-ABC^+^/CD80^+^/CD83^+^/CD86^+^/CD40^+^/CCR7^+^). They were then cryopreserved until the day of administration.

### Treatment

The DC suspension was adjusted to a total volume of 1.0 mL using saline. All patients were intradermally injected 5–7 times with DCs (approximately 10^7^ cells/injection) in close proximity to the axial and/or inguinal lymph nodes. Injections were repeated every 14–21 days. OK-432, a streptococcal immunological adjuvant, was administered simultaneously with the DC vaccine to patients without serious allergies to penicillin or other drugs. Tolerable doses of OK-432 ranged from 0.5 to 5 KE, and the appropriate doses were determined according to the incidence of fever after administration.

### Immunological assessment

Immunological function was found to be associated with survival on DC vaccine administration. For the functional analysis of cancer immunotherapy, CD4^+^ T cells, CD8^+^ T cells, and natural killer (NK) cells were obtained from blood samples before and after DC vaccine administration. Cells with a CD16^+^/CD56^+^ phenotype as determined by flow cytometry were considered to be NK cells.

### Tetramer staining

WT1-specific cytotoxic T lymphocytes (CTLs) were also evaluated for adequate induction. The frequency of WT1-specific CTLs in each patient was determined with either WT1-HLA-A*2402 or 0201 tetramers by flow cytometry analysis as previously described [18]. Briefly, T cells were incubated with Clear Back (MBL, Nagoya, Japan) prior to tetramer staining to block the Fc receptors. They were then stained with phycoerythrin-labeled HLA-A*2402 WT1 mutated (CYTWNQMNL) and HLA-A*0201 WT1 wild-type (RMFPNAPYL) tetramers (MBL), followed by fluorescein isothiocyanate-labeled anti-human CD3, CD4, and CD8. The staining was performed at 4°C for 30 minutes, and the cells were washed twice before flow cytometry analysis.

### Evaluation

All adverse events (AEs) were graded and documented according to the Common Terminology Criteria for Adverse Events, version 4.0. Clinical assessments were performed at baseline, 3 months, and 6 months via computed tomography (CT) or magnetic resonance imaging (MRI). Response was expressed as the proportion of patients with a complete response (CR), partial response (PR), stable disease (SD), or progressive disease (PD), as well as disease control rate (DCR) or objective response rate (ORR), as assessed according to the Response Evaluation Criteria in Solid Tumors (RECIST) version 1.1. We assessed transient erythema in the patients’ forearm skin within 24–48 h after vaccination. Fever after vaccination was also assessed and defined as a body temperature of ≥38°C at 48 hours after vaccination. In addition, we used the neutrophil-to-lymphocyte ratio (NLR) as a simple index of systemic inflammation [20].

### Statistical analyses

The Kaplan-Meier probability estimates of overall survival (OS) were calculated, and statistical differences between the treatment arms were determined using the log-rank test. A multivariate analysis was performed using the Cox regression method to estimate the hazard ratios (HRs) with a 95% confidence interval (CI). Categorical data were compared using the Fisher exact probability test or the chi-square test. Differences were considered statistically significant when P values were <0.05.

## Results

### Patients

A total of 71 patients who initially received chemotherapy for ROC followed by DC-based immunotherapy were included in our study. Of these, 8 patients who received less than 5 rounds of the DC vaccine and 5 who were unavailable for follow-up examinations were excluded. Two patients who received the DC vaccine pulsed with peptides eluted from autologous OCs were also excluded. Thus, the final analysis included 56 eligible patients whose clinicopathological characteristics are summarized in Table 1. The patients’ age ranged from 23 to 70 years (median, 55.0 years). Histological diagnoses included serous cystadenocarcinoma (n = 37), endometrioid adenocarcinoma (n = 6), clear cell adenocarcinoma (n = 5), others (n = 4, Additional file 1: Table S2), and unknown (n = 4). Forty-six patients received DCs pulsed with WT1 (WT1 only, n = 7; WT1 + MUC1, n = 31; WT1 + MUC1 + CA125, n = 3; and WT1 + CA125, n = 5), whereas the others received DCs pulsed with MUC1 and/or CA125. All patients in this study had initially received chemotherapy for ROC. Of these, 27 (48%) received platinum-based chemotherapy while the others (52%) received non-platinum-based chemotherapy or received no chemotherapy during DC vaccination.

**Table 1:** Patient characteristics

	**Overall**	**Albumin ≥ 4.0**	**Albumin < 4.0**	**P value**
Number of patients	56	36	20	
Age (year)				0.6563
Median (range)	55 (23–70)	52 (23–70)	56 (28–68)	
ECOG performance status score – no. (%)				0.0877
0 -1	48 (86)	33	15	
2-	8 (14)	3	5	
Histological diagnosis – no. (%)				0.2583
Serous cystadenocarcinoma	37 (66)	25	12	
Endometrioid adenocarcinoma	6 (11)	5	1	
Clear cell adenocarcinoma	5 (8.9)	2	3	
Others	4 (7.1)	3	1	
Unknown	4 (7.1)	1	3	
Site of metastasis – no. (%)				
Peritoneum	29 (52)	20	9	0.1413
Liver	11 (20)	7	4	0.5185
Ascites	21 (38)	13	8	0.4371
Peptide				0.1694
WT1	46 (82)	31	15	
MUC1	39 (70)	27	12	
CA125	16 (29)	7	9	
Number of DC vaccine administration				
Median (range)	7 (5–20)	7 (5–18)	7 (5–20)	0.6867

### Toxicity and AEs

The AEs were tolerable in all patients. No serious acute allergic reaction such as anaphylaxis was observed. The most common AEs were injection site reaction (68%) and fever (32%). Other common AEs such as arthralgia and elevated liver enzyme levels were not observed. No grade 3–4 toxicity or evidence of autoimmune sequelae was documented.

### Immunological assessment

**Figure 1:**
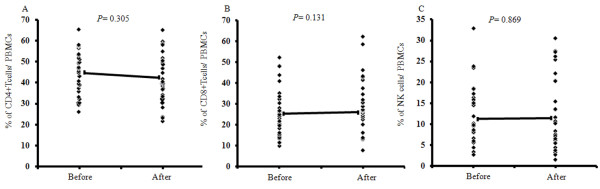
**Frequencies of CD4+ T cells, CD8**^**+**^**T cells, and natural killer cells in patients before and after dendritic cell-based immunotherapy targeting synthesized peptides for recurrent ovarian cancer.(A)** Data on frequencies of CD4+ T cells (n = 31), **(B)** CD8^+^ T cells (n = 31), and **(C)** natural killer cells (n = 31) are expressed as a percentage of peripheral blood mononuclear cells.

**Figure 2:**
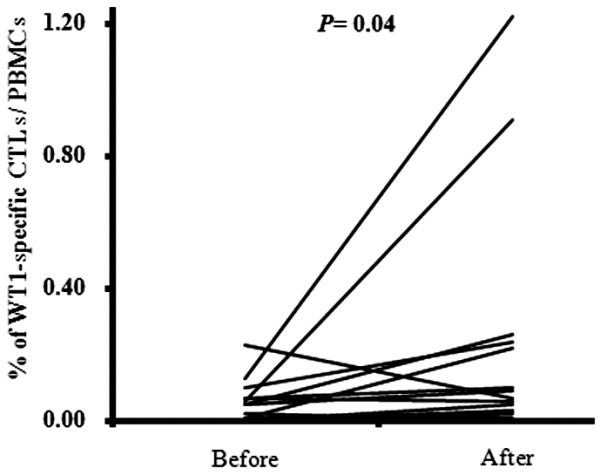
**Frequency of Wilms tumor (WT) 1-specific cytotoxic T lymphocytes (CTLs) in patients before and after therapy.** WT1-specific CTLs were detected using WT1 tetramers in 17 patients. Data are expressed as a percentage of peripheral blood mononuclear cells.

Fever and erythema after vaccination and frequencies of CD4^+^ T cells, CD8^+^ T cells, and NK cells were examined to assess immunological function. However, no remarkable changes were observed in CD4+ T cell, CD8^+^ T cell, and NK cell frequencies after vaccination (Figure 1A–C). In addition, none of these factors affected the median survival time (MST). Forty-six of the 56 patients received DCs pulsed with WT1 peptide, 17 of whom were evaluable for WT1-specific CTLs. The frequency of WT1-specific CTLs increased in 12 of these examined patients (70.6%; P = 0.04; Figure 2 and Additional file 1: Table S2). However, no significant differences in clinical outcomes were observed between patients with WT1-specific CTL increase and those without such an increase.

### Clinical outcomes

**Figure 3:**
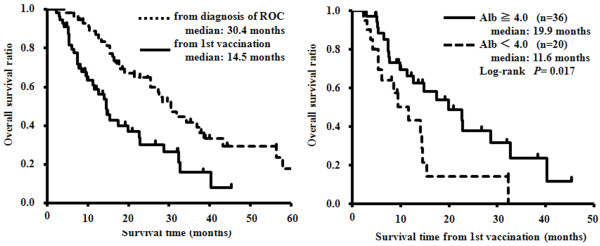
**Kaplan-Meier survival curves for the patients receiving dendritic cells-based vaccination.** (Left) Survival curve from diagnosis (dotted line) and from the first vaccination (solid line). (Right) Comparison of the overall survival rates according to albumin levels (≥4.0 g/dL [solid line] and <4.0 g/dL [dotted line]).

Clinical responses were evaluated in 56 patients. The MST from diagnosis was 30.4 months and that from the first vaccination was 14.5 months (Figure 3, left). The 1- and 2-year survival rates from diagnosis were 87% and 65%, respectively. Therapeutic efficacy was evaluated according to RECIST in all 56 patients at 3 months after the first vaccination, and none of the patients showed CR. However, 2 patients (3.6%) achieved PR, 14 (25%) had SD, 32 (57%) had PD, and 8 (14%) were not evaluated. The DCR and ORR were 29% and 3.6%, respectively (Table 2). At the time of the final analysis, 35 patients (63%) had died of cancer. Multivariate analysis revealed that an albumin level of ≥4.0 g/dL and a lactate dehydrogenase (LDH) level of <200 IU/L before vaccination were significantly associated with the MST from the first vaccination (Table 3). The albumin level before vaccination was a significant factor for MST prolongation, as determined by the log-rank test (P = 0.017; HR = 0.46; 95% CI, 0.18–0.85) and multivariate analysis. The MST from the first vaccination in patients with albumin levels of ≥4.0 and <4.0 g/dL were 19.9 and 11.6 months, respectively (Figure 3, Right). The DCR and ORR were 36% and 5.5%, respectively, in the 36 (64%) patients with albumin levels of ≥4.0 g/dL. In contrast, these rates were 15% and 0%, respectively, in patients with albumin levels of <4.0 g/dL (Table 2).

**Table 2:** Clinical response to the DC vaccine

	**3 months**						**6 months**					
**Clinical response**	**All patients**		**Alb ≥ 4.0**		**Alb < 4.0**		**All patients**		**Alb ≥ 4.0**		**Alb < 4.0**	
	***N***	***%***	***N***	***%***	***N***	***%***	***N***	***%***	***N***	***%***	***N***	***%***
CR	0	0	0	0	0	0	0	0	0	0	0	0
PR	2	3.6	2	5.5	0	0	1	1.8	1	2.8	0	0
SD	14	25	11	30.6	3	15	7	12.5	5	13.9	2	10
PD	32	57.1	19	52.8	13	65	42	75	27	75	15	75
NE	8	14.3	4	11.1	4	20	6	10.7	3	8.3	3	15
Total	56	100	36	100	20	100	56	100	36	100	20	100
ORR*	2	3.6	2	5.5	0	0	1	1.8	1	2.8	0	0
DCR**	16	28.6	13	36.1	3	15	8	14.3	6	16.7	2	10

*Objective response rate (ORR), considers CR and PR.

**Disease control rate (DCR), considers CR, PR, and SD.

**Table 3:** Multivariate analysis of prognostic factors in ovarian cancer (n = 56)

**Variable**		**Log-rank**	**Wilcoxon**	**Cox’s hazard regression**		
				**Hazard ratio**	**95% CI**	***P*** **value**
Hemoglobin (g/dL)	≥11	0.131	0.165	1	0.524-5.356	0.385
	<11			1.675		
Albumin (g/dL)	≥4.0	0.017	0.022	1	1.055-11.34	0.04
	<4.0			3.459		
LDH (IU/L)	≥200	0.107	0.131	1	0.077-0.975	0.046
	<200			0.274		
CRP (mg/dL)	<0.5	0.009	0.01	1	0.757-13.47	0.114
	≥0.5			3.192		
Lymphocytes (/μL)	≥1,200	0.688	0.499	1	0.66-7.527	0.197
	<1,200			2.229		
Neutrophil (/μL)	<4,000	0.766	0.853	1	0.291-4.046	0.903
	≥4,000			1.085		
Peritoneal metastasis	Yes	0.273	0.483	1	0.137-1.038	0.059
	No			0.378		
Liver metastasis	Yes	0.795	0.603	1	0.236-5.759	0.851
	No			1.165		
Ascites	Yes	0.388	0.277	1	0.593-4.84	0.325
	No			1.694		
Fever after DC vaccine (°C)	<38	0.657	0.72	1	0.235-3.549	0.896
	≥38			0.913		
Erythema (mm)	≥30	0.083	0.014	1	0.647-7.73	0.203
	<30			2.236		
WT1 peptide	Yes	0.959	0.608	1	0.388-13.65	0.359
	No			2.3		
MUC1 peptide	Yes	0.479	0.59	1	0.324-2.752	0.917
	No			0.945		

We used an NLR cutoff of 4 to evaluate the MST from the first DC vaccine and found that the NLR was significantly correlated with the MST by using the log-rank test (P = 0.02; HR = 2.16; 95% CI, 1.15–5.97). The MST of patients with an NLR of <4 was significantly longer than that of patients with an NLR of ≥4 (19.9 vs. 9.5 months, Additional file 2). The DCR was 35% in patients with an NLR of <4 compared to 13% in those with an NLR of ≥4 (Additional file 1: Table S1). Neither the use of OK-432 nor the amount of OK-432 administered was significantly associated with survival, indicating that OK-432 itself did not affect the survival of patients with ROC (log-rank test, P = 0.854, 14.5 vs. 19.9 months; Wilcoxon, P = 0.498).

## Discussion

Although some clinical trials of DC immunotherapy for OC have been conducted, most of these were designed with small sample sizes (approximately 11–22 patients) and included patients with different stages of OC [8-10]. Differences in biological behavior and individual disease burden were some of the issues encountered when comparing the efficacy of DC-based immunotherapy in heterogeneous patient populations, including patients with recurrent, unresectable, and metastatic disease. The present study was therefore designed to include only ROC patients who had received prior chemotherapy (Additional file 1: Table S2) in order to accurately assess the therapeutic effects and evaluate alternative approaches. Despite its retrospective design and small sample size, the present study yielded 3 major findings in ROC patients receiving the DC vaccine. First, DC-based immunotherapy was well tolerated in all patients, with no serious complications observed. Second, DC administration may be adequate for generating immune responses. Finally, according to multivariate analysis, albumin levels of ≥4.0 g/dL and LDH levels of <200 IU/L before vaccination were significant independent factors for an improved MST.

In this study, the frequency of WT1-specific CTLs increased in 12 (70.6%) of the 17 patients who were examined using the WT1-specific CTL tetramer assay. We found that an increase in the frequency of WT1-specific CTLs was not significantly associated with MST. In addition, an increase in the frequency of WT1-specific CTLs did not affect CD8^+^ T cells. Thus, an increase in antigen-specific CTLs did not appear to directly contribute to the MST. Similarly, although vaccine therapies developed to date have demonstrated immunological responses, only minor clinical benefits have been observed [8,9,21].

ROC patients with initially platinum-sensitive disease usually receive platinum-based regimens, even as second-line chemotherapy. Their response rates range from 31% to 66%, with a MST of 17–29 months [22,23]. In contrast, the MST of platinum-resistant patients is only 10.6 months [5]. In this study, the MST from diagnosis was 30.4 and that from the first vaccination was 14.5 months. DC-based immunotherapy was elected by the patients themselves without our recommendations, leading to inconsistencies in the date of DC-based immunotherapy initiation. Therefore, although our results suggest that DC immunotherapy might prolong survival time, we could not adequately address the differences in survival benefits between our study and previous ones because of the differences in starting points for survival time calculation, patient population, anticancer drugs, and therapy administration schedules. A future prospective study with a larger sample size is thus needed to confirm and validate our findings. The clinical design of such a study should include 4 independent prospective trials to compare chemotherapy platinum-sensitive +/-DC-immunotherapy and platinum-resistant +/-DC-immunotherapy in a controlled ROC patient population. Since DC-based immunotherapy has been reported to show late efficacy [24], the appropriate endpoint should be overall survival, regardless of the study sample size.

The multivariate Cox proportional hazard analysis identified albumin and LDH levels as significant independent factors. Albumin levels are associated with cachexia and ascites, leading to malnutrition in ROC patients. The nutritional status of a cancer patient, which can be assessed by the serum albumin level, is known to be associated with survival. Asher et al. [25] clearly demonstrated that a low serum albumin level was associated with poor survival and that the albumin level could be an independent prognostic predictor of survival in OC patients. In our previous study, low albumin levels were responsible for poor prognosis in patients receiving DC immunotherapy for biliary tract cancers [19], and the same phenomenon was observed in this study on ROC patients. In both studies, an albumin level of ≥4 g/dL seemed to indicate suitability for DC immunotherapy, and the nutritional status was found to be a promising factor for promoting therapeutic effects.

LDH is a major enzyme in glycolysis that reversibly catalyzes the conversion of pyruvate to lactic acid. Boran et al. [26] showed that high serum LDH levels are linked to a poor prognosis in patients with OC. There could be several reasons for the ominous prognostic significance of serum LDH in malignancies. For example, an acidic extracellular pH has been shown to activate gelatinase activity and cathepsin D production, which helps increase the invasiveness of cancer cells [27,28]. Additionally, the lactate-mediated activation of macrophage-associated angiogenesis might also facilitate metastasis [29]. Finally, a low pH protects the mitochondria from oxidative stress and could account for the increased resistance of cancer cells to hypoxia-induced apoptosis [30]. Further investigation is required to determine whether the abovementioned or any additional mechanisms underlie the strong and independent association of LDH with the prognosis of ROC patients.

Inflammation plays a critical role in the pathogenesis and progression of cancer. Recently, the derived NLR has been shown to influence the clinical outcomes of various cancer types, including ROC [20]. The number of neutrophils might reflect the levels of circulating angiogenesis-regulating chemokines, growth factors, and proteases, which are major contributors to tumor-related angiogenesis [31]. In addition, lymphocytes are involved in the production of cytokines that inhibit the proliferation and metastasis of tumor cells [32]. Therefore, the NLR could act as a marker of the balance between the host inflammatory and immune responses. In our study, patients with a high NLR had a poor prognosis, suggesting that the NLR might be another predictor of the MST in ROC patients.

A number of cancer-associated gene products evoke immune recognition, but host reactions rarely impede disease progression. A strong therapeutic effect has not been confirmed in ROC patients. The weak immunogenicity of nascent tumors contributes to such a failure by the host defense. Our next option to treat ROC patients with DC immunotherapy is the concomitant use of either antagonists of immune-repressor molecules or agonists of immune-activating receptors such as checkpoint blockade receptors comprising cytotoxic T-lymphocyte associated antigen 4 (CTLA-4) (anti-CTLA-4 antibody, ipilimumab; Bristol-Myers-Squibb, NY, USA) and programmed death-1 (PD-1) (anti-PD-L1 monoclonal antibody therapy, BMS-936559). These drugs serve to accelerate antitumor immune responses and improve the therapeutic effect potential in OC [33,34]. The combination of immunomodulatory properties with DC-based immunotherapy targeting synthesized peptides might help prolong the survival of ROC patients.

## Abbreviations

OC: Ovarian cancer; ROC: Recurrent ovarian cancer; DC: Dendritic cell; WT1: Wilms tumor 1; MUC1: Mucin 1 cell surface associated; CA125: Cancer antigen 125; IHC staining: Immunohistochemical staining; MST: Median survival time; ORR: Objective response rate; DCR: Disease control rate; RECIST: Response evaluation criteria in solid tumors; NLR: Neutrophil to lymphocyte ratio; IL: Interleukin; LDH: Lactate dehydrogenase; CRP: C-reactive protein; CTLs: cytotoxic T lymphocytes; CTLA4: Cytotoxic T-lymphocyte–associated antigen 4; PD-1: Programmed death-1.

## Competing interests

The authors have no financial or personal relationships with people or organizations that could inappropriately influence this work.

## Authors’ contributions

MK, AC, and MN conceived and designed the study and collected, assembled, analyzed, and interpreted the data. EY provided study materials. HI, YS, SS, MO, YY, and NS analyzed and interpreted the data. MK and MN wrote the manuscript and approved the final manuscript. All authors read and approved the final manuscript.

## Supplementary Material

Additional file 1: Table S1 Clinical response to the DC vaccine according to neutrophil-to-lymphocyte ratio. **Table S2.** Patient demographics, treatment characteristics, and immunological responses. Click here for file

Additional file 2 Comparison of the overall survival rates according to the neutrophil-to-lymphocyte ratio (<4 [solid line] and ≥4 [dotted line]). Click here for file
