# Supplementary material for: The feasibility and clinical effects of dendritic cell-based immunotherapy targeting synthesized peptides for recurrent ovarian cancer
Source: J Ovarian Res. 2014 May 7;7:48. doi: 10.1186/1757-2215-7-48 (PMC4108140; doi:10.1186/1757-2215-7-48)
Supplement: Additional file 1: Table S1 — Clinical response to the DC vaccine according to neutrophil-to-lymphocyte ratio. Table S2. Patient demographics, treatment characteristics, and immunological responses. [file 1757-2215-7-48-S1.docx]

Additional file 1: Table S1. Clinical response to the DC vaccine according to neutrophil-to-lymphocyte ratio.

|  | 3 months | | | | 6 months | | | |
| --- | --- | --- | --- | --- | --- | --- | --- | --- |
| Clinical | NLR ≥ 4.0 | | NLR < 4.0 | | NLR ≥ 4.0 | | NLR < 4.0 | |
| response | *N* | *%* | *N* | *%* | *N* | *%* | *N* | *%* |
| CR | 0 | 0 | 0 | 0 | 0 | 0 | 0 | 0 |
| PR | 0 | 0 | 2 | 5 | 0 | 0 | 1 | 2.5 |
| SD | 2 | 12.5 | 12 | 30 | 2 | 12.5 | 5 | 12.5 |
| PD | 12 | 75 | 20 | 50 | 12 | 75 | 30 | 75 |
| NE | 2 | 12.5 | 6 | 15 | 2 | 12.5 | 4 | 10 |
| Total | 16 | 100 | 40 | 100 | 16 | 100 | 40 | 100 |
| ORR | 0 | 0 | 2 | 5 | 0 | 0 | 1 | 2.5 |
| DCR | 2 | 12.5 | 14 | 35 | 2 | 12.5 | 6 | 15 |

Neutrophil-to-lymphocyte ratio [NLR]

Objective response rate [ORR] (considers CR and PR), Disease control rate [DCR] (considers CR, PR, and SD).

Additional file 1: Table S2. Patient demographics, treatment characteristics, and immunological responses.

| Patient No. | Age | Diagnosis | Type of treatment before recurrence | Vaccination peptides | Serum albumin (g/dl) | Immunological responses | | | | | | | | Survival time from 1st vaccination (months) |
| --- | --- | --- | --- | --- | --- | --- | --- | --- | --- | --- | --- | --- | --- | --- |
|  |  |  |  |  |  | CD4 (%) | | CD8 (%) | | NK (%) | | WT1 (%) | |  |
|  |  |  |  |  |  | Before | After | Before | After | Before | After | Before | After |  |
| 1 | 44 | SCC | TC, PTX, CDDP, CPT-11, PLD, DTX | WT1, MUC1, CA125 | 3.9 | 35.9 | 38.4 | 35.1 | 30.4 | 14.6 | 10.2 |  |  | 11.6 |
| 2 | 48 | SCC | TC, CPT-11, NDP, RT | WT1, MUC1, CA125 | 4.3 |  |  |  |  |  |  |  |  | 7.5 |
| 3 | 70 | SCC | TC, GEM | WT1, MUC1 | 4.1 | 32.2 | 32.5 | 43.7 | 42.7 | 8.6 | 7.2 | 0 | 0.05 | 7.7 |
| 4 | 55 | SCC | TC, CPT-11, PLD, GEM | WT1, MUC1 | 2.7 | 26.1 | 23.8 | 32.1 | 34.6 | 9.5 | 5.6 |  |  | 4.7 |
| 5 | 69 | SCC | TC, PTX, CPT11, TPT | WT1, MUC1 | 4.0 | 39.1 | 37.3 | 26.7 | 24.3 | 10.1 | 15.4 | 0.02 | 0 | 14.5 |
| 6 | 55 | SCC | TC | WT1, MUC1 | 4.2 | 38.7 | 39.5 | 25 | 27.1 | 11.9 | 10.2 | 0 | 0 | 19.1 |
| 7 | 51 | SCC | TC | WT1, MUC1 | 4.2 | 58.1 | 58.2 | 25 | 25.6 | 6.1 | 3.8 |  |  | 9.1 |
| 8 | 66 | SCC | TC, CPT11+MMC, PLD | WT1, MUC1 | 3.6 | 45.6 | 36.5 | 21.6 | 27 | 18.6 | 20.4 | 0.06 | 0.91 | 6.1 |
| 9 | 49 | SCC | TC, PLD+CBDCA | WT1, MUC1 | 4.1 | 49.6 | 28.3 | 19.6 | 43.3 | 8.7 | 11.6 |  |  | 32.0 |
| 10 | 55 | SCC | TC, CPT-11, GEM, PLD, TPT | WT1, MUC1 | 4.2 | 51.1 | 59.5 | 14.5 | 13.3 | 5.6 | 2.7 | 0.01 | 0.01 | 12.6 |
| 11 | 55 | SCC | TC | WT1, MUC1 | 4.9 | 57.4 | 58.7 | 13.9 | 13.8 | 8 | 8.3 |  |  | 13.7 |
| 12 | 58 | SCC | TC, PLD, CPT-11, GEM | WT1, MUC1 | 4.2 | 56.4 | 59.6 | 13.2 | 12.9 | 9.8 | 13.5 | 0.01 | 0.22 | 12.2 |
| 13 | 59 | SCC | TC | WT1, MUC1 | 4.3 | 37.5 | 54.9 | 11.3 | 25.7 | 9.8 | 7.5 | 0.01 | 0.02 | 26.7 |
| 14 | 44 | SCC | DC | WT1, MUC1 | 4.4 | 57.9 | 50.5 | 10 | 7.7 | 16.3 | 27.3 |  |  | 9.6 |
| 15 | 53 | SCC | TC, CPT-P | WT1, MUC1 | 4.3 |  |  |  |  |  |  |  |  | 38.4 |
| 16 | 58 | SCC | TC | WT1, MUC1 | 4.2 |  |  |  |  |  |  |  |  | 5.1 |
| 17 | 48 | SCC | TC, CPT-P, NDP | WT1, MUC1 | 4.4 |  |  |  |  |  |  |  |  | 40.3 |
| 18 | 59 | SCC | CPT-11 | WT1, MUC1 | 4.0 |  |  |  |  |  |  |  |  | 17.5 |
| 19 | 55 | SCC | TC, NDP | WT1, MUC1 | 3.5 |  |  |  |  |  |  |  |  | 3.7 |
| 20 | 64 | SCC | TC, PT | WT1, MUC1 | 4.3 |  |  |  |  |  |  |  |  | 4.9 |
| 21 | 64 | SCC | TC, CBDCA, PLD | WT1, MUC1 | 3.0 |  |  |  |  |  |  | 0 | 0.01 | 14.5 |
| 22 | 63 | SCC | TC, PLD | WT1, MUC1 | 4.4 |  |  |  |  |  |  |  |  | 2.9 |
| 23 | 50 | SCC | CDDP, TC, CPT-P, CAP | WT1, CA125 | 2.8 | 46.7 | 51.8 | 47.9 | 41.5 | 14.8 | 25.4 | 0.13 | 1.22 | 9.5 |
| 24 | 52 | SCC | TC | WT1, CA125 | 4.7 | 57.4 | 50.7 | 33.6 | 37.4 | 4.4 | 10.4 |  |  | 14.9 |
| 25 | 57 | SCC | TC | WT1, CA125 | 4.4 | 65.5 | 33.5 | 30.3 | 62.2 | 8.1 | 3.4 | 0.1 | 0.24 | 32.8 |
| 26 | 50 | SCC | TC | WT1 | 4.3 | 29.2 | 21.8 | 26.6 | 31.9 | 23.5 | 30.5 | 0 | 0.02 | 22.6 |
| 27 | 60 | SCC | TC, PLD, CPT-11, RT | WT1 | 3.7 | 49.2 | 36.8 | 15.3 | 16.1 | 14.5 | 10.5 | 0.07 | 0.1 | 14.1 |
| 28 | 55 | SCC | TC, CPT-11, PLD, GEM | WT1 | 4.2 |  |  |  |  |  |  |  |  | 5.1 |
| 29 | 42 | SCC | TC | WT1, CA125 | 4.0 |  |  |  |  |  |  |  |  | 22.8 |
| 30 | 56 | SCC | PTX, DTX, CPT-11, GEM, PLD | WT1, CA125 | 3.9 |  |  |  |  |  |  |  |  | 32.3 |
| 31 | 64 | SCC | TC, DC, CPT-P | MUC1, CA125 | 3.6 |  |  |  |  |  |  |  |  | 14.5 |
| 32 | 56 | SCC | TC | MUC1, CA125 | 4.1 |  |  |  |  |  |  |  |  | 45.4 |
| 33 | 47 | SCC | TC, CPT-P, GEM | MUC1, CA125 | 3.8 |  |  |  |  |  |  |  |  | 8.2 |
| 34 | 28 | SCC | TC | MUC1 | 4.1 |  |  |  |  |  |  |  |  | 19.9 |
| 35 | 45 | SCC | TC | CA125 | 3.8 | 47.4 | 65.2 | 48.3 | 26.6 | 5.7 | 6.7 |  |  | 9.3 |
| 36 | 63 | SCC | CAP, TC, RT | CA125 | 2.9 |  |  |  |  |  |  |  |  | 2.3 |
| 37 | 65 | SCC | CDDP+ MMC | CA125 | 4.4 |  |  |  |  |  |  |  |  | 6.6 |
| 38 | 47 | EAC | TC, CPT-11, DC | WT1, MUC1 | 4.4 | 51.8 | 39.9 | 24.9 | 28.7 | 3.4 | 4.4 |  |  | 5.4 |
| 39 | 47 | EAC | TC, AP | WT1, MUC1 | 4.2 | 39.3 | 41.8 | 20.7 | 20.1 | 17.2 | 6.5 |  |  | 4.8 |
| 40 | 46 | EAC | Phenylbutyrate, RT | WT1, MUC1 | 4.5 | 29.4 | 23.2 | 20.2 | 22.5 | 15.1 | 20.3 | 0.23 | 0.07 | 11.3 |
| 41 | 63 | EAC | TC, PLD | WT1, MUC1 | 4.3 | 53 | 44.8 | 11.7 | 13 | 8.3 | 26.2 | 0.05 | 0.09 | 28.7 |
| 42 | 63 | EAC | DC, PLD | WT1, MUC1 | 3.7 |  |  |  |  |  |  |  |  | 5.4 |
| 43 | 47 | EAC | TC, VP-16 | WT1 | 4.4 |  |  |  |  |  |  |  |  | 7.4 |
| 44 | 36 | CCA | TC, CPT-11, PLD | WT1, MUC1, CA125 | 1.2 |  |  |  |  |  |  |  |  | 3.1 |
| 45 | 39 | CCA | TC | WT1, MUC1 | 4.3 | 49.7 | 48.7 | 32.6 | 30.7 | 2.7 | 1.5 |  |  | 7.4 |
| 46 | 56 | CCA | TC, CPT-P, GEM | WT1, MUC1 | 3.9 | 43.1 | 39.6 | 16.1 | 20.2 | 15.9 | 13.6 | 0 | 0.03 | 8.6 |
| 47 | 35 | CCA | CPT-P | WT1, MUC1 | 3.4 |  |  |  |  |  |  |  |  | 5.4 |
| 48 | 51 | CCA | CPT-P, RT | MUC1 | 4.4 | 30.4 | 30.2 | 23.1 | 25.5 | 32.9 | 27.3 |  |  | 21.0 |
| 49 | 58 | Others* | TC, DC, CPT-11 | WT1, MUC1 | 4.1 |  |  |  |  |  |  |  |  | 14.8 |
| 50 | 23 | Others** | TC | WT1, MUC1 | 4.0 | 40.8 | 34.3 | 52.2 | 58.4 | 15.2 | 10.7 | 0.07 | 0.06 | 9.9 |
| 51 | 52 | Others^#^ | VP-16+CDDP, TC, RT | WT1 | 4.8 | 44.6 | 47 | 24.1 | 26.3 | 6.6 | 6.1 |  |  | 10.1 |
| 52 | 28 | Others^##^ | TC, RT | WT1 | 2.4 | 46.9 | 46.8 | 40.9 | 46.1 | 9.8 | 3.2 | 0.05 | 0.26 | 15.4 |
| 53 | 57 | Unknown | TC | WT1, MUC1 | 3.7 | 33.3 | 49.3 | 28.1 | 24.3 | 18.5 | 5.5 |  |  | 8.2 |
| 54 | 68 | Unknown | TC | WT1 | 3.8 | 30.3 | 31.8 | 18.2 | 23.4 | 18.3 | 22.1 |  |  | 32.2 |
| 55 | 39 | Unknown | TC, CPT-11 | CA125 | 3.7 |  |  |  |  |  |  |  |  | 4.1 |
| 56 | 45 | Unknown | TC | CA125 | 4.2 |  |  |  |  |  |  |  |  | 5.2 |

Serous cystadenocarcinoma [SCA], Endometrioid adenocarcinoma [EAC], Clear cell adenocarcinoma [CCA], Doxorubicin and cisplatin [AP], Cyclophosphamide, doxorubicin（adriamycin）, and cisplatin [CAP], Carboplatin [CBDCA], Cisplatin [CDDP], Irinotecan and cisplatin [CPT-P], Irinotecan [CPT-11], Docetaxel and carboplatin [DC], Docetaxel [DTX],Gemcitabine, [GEM], Mitomycin C [MMC], Nedaplatin [NDP], Pegylated liposomal doxorubicin [PLD], Paclitaxel [PTX], Paclitaxel and carboplatin [TC], Paclitaxel and cisplatin [PT], Nogitecan [TPT], Etoposide [VP-16], Radiation therapy [RT]. *EAC +CCA, ** Malignant transformation of dermoid cyst(squamous cell carcinoma),

^#^ Cystadenocarcinoma, ^##^ Mature teratoma with squamous cell carcinoma transformation.
