# Supplementary figures and images for: The feasibility and clinical effects of dendritic cell-based immunotherapy targeting synthesized peptides for recurrent ovarian cancer
Source: J Ovarian Res. 2014 May 7;7:48. doi: 10.1186/1757-2215-7-48 (PMC4108140; doi:10.1186/1757-2215-7-48)

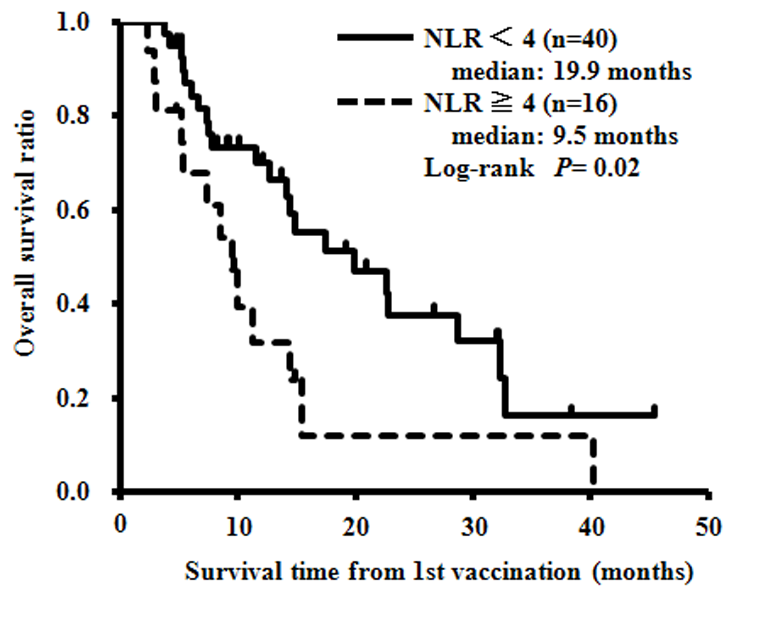

Supplement: Additional file 2 — Comparison of the overall survival rates according to the neutrophil-to-lymphocyte ratio (<4 [solid line] and ≥4 [dotted line]). [file 1757-2215-7-48-S2.tiff]
